# Feeding Lactic Acid Bacteria with Different Sugars: Effect on Exopolysaccharides (EPS) Production and Their Molecular Characteristics

**DOI:** 10.3390/foods12010215

**Published:** 2023-01-03

**Authors:** Andrea Fuso, Elena Bancalari, Vincenzo Castellone, Augusta Caligiani, Monica Gatti, Benedetta Bottari

**Affiliations:** Department of Food and Drug, University of Parma, 43124 Parma, Italy

**Keywords:** Lactic acid bacteria, EPS, impedometric analysis, EPS molecular characterization, GC-MS, HPSEC-RID

## Abstract

Exopolysaccharides (EPS) are complex molecules produced by some microorganisms and used in foods as texturizers and stabilizers, their properties depending on their chemical structure. In this work, three different lactic acid bacteria (LAB), were tested for their ability to produce EPS, by using five different mono- and disaccharides as their sole carbon source. The growth and acidifying ability were analysed, the EPSs were quantified by the official method AOAC 991.43, and their chemical structure was investigated. The amount of EPS varied from 0.71 g/L to 2.38 g/L, and maltose was the best sugar for EPS production by *Lacticaseibacillus paracasei* 2333. *Lacticaseibacillus rhamnosus* 1019 produced the highest amount when fed with lactose, whereas the EPS amount of *Lactobacillus bulgaricus* 1932 was not significantly different depending on the sugar type. The EPS chains consisted of fructose, galactose, glucose, mannose, ribose, glucosamine, galactosamine, and in some cases rhamnose in different proportions, depending on the strain and carbon source. The molecular weight of EPS ranged from <10 KDa to >500 KDa and was again highly dependent on the strain and the sugar used, suggesting the possibility of growing different strains under different conditions to obtain EPS with different potential applications in the food system.

## 1. Introduction

Lactic acid bacteria (LAB) have been closely associated with humans since ancient times and throughout history [1]. Nowadays, LAB are receiving more and more attention due to their ability to ferment various matrices and produce healthy compounds with high value. Among them, exopolysaccharides (EPS) are gaining interest due to their technological and nutritional functionality [2,3]. Different LAB species can produce a wide variety of EPS with different structures, sizes and thus, different functions [4,5]. Some recent studies [6,7,8] have highlighted the prebiotic effect of EPS, which increases the amount of desired microorganisms in the intestine, especially in the colon. Other authors have recently reviewed the industrial application of EPS, highlighting their effect on the texture and rheological properties of certain fermented products where they were produced in situ. In particular, they investigated how the chemical properties of EPS affect the interactions with milk protein in fermented dairy products [9].

In general, EPS can be divided into two macro-categories depending on the composition of the saccharide chain: (i) heteropolysaccharides (HePS) and (ii) homopolysaccharides (HoPS). HoPS are usually composed of the same monosaccharide and have a linear and larger structure (>10^6^ Da) than HePS [4,10]. These two types of polysaccharides also differ in terms of the biosynthetic pathway. HoPS are usually synthesised outside the cell, where a specific enzyme collects and assembles the monomeric sugar residues [10]. HoPS are not charged and are mainly associated with prebiotic effects [6,8,11]. HePS consist of two or more sugar moieties in a linear or branched chain. HePS are smaller (10^4^–10^6^ Da), may have non-carbohydrate residues in their composition, including charged groups, and are primarily associated with enhancing effects on host immune functions [8,11]. The synthetic pathway of HePS is more complex compared to HoPS, as they are normally synthesized within the cell and then carried out of the cell. EPS are essential components of extracellular polymeric substances, and it has been reported that they can be used by cells as a strategy to cope with nutrient deficiency or harsh conditions, such as pollution or the presence of harmful substances [10]. Approximately 30 different LAB species are recognized as EPS producers, the best known being: *Lacticaseibacillus paracasei, Lacticaseibacillus rhamnosus*, *Lactobacillus helveticus*, *Lactobacillus delbrueckii*, *Lactobacillus acidophilus*, *Latilactobacillus sakei* and *Lactiplantibacillus plantarum* [7,12,13,14,15,16,17,18]. Many of these EPS producers have been isolated from traditional food matrices, where they help to improve the texture, mouthfeel, and stability of fermented products. As a result, EPS have attracted much attention in recent years due to their promising properties that can be exploited to improve food technological characteristics, but also to increase nutritional value [19,20]. The industrial production of EPS is mainly achieved by feeding LAB with food by-products and media-rich in sucrose [21]. However, some studies have demonstrated that LAB are able to adapt to different media and drive the EPS production and molecular composition depending on the medium [22,23]. Midik and colleagues suggested glucose as the best solution to increase EPS production by different LAB, while on the other hand fructose reduced EPS production compared to control media [24]. In another study, *L. delbrueckii* subsp. *bulgaricus* NCFB (National Collection of Food Bacteria, originally located in Shinfield, Reading, Berkshire, UK) 2722 was shown to produce a higher amount of EPS when grown in media containing glucose or lactose, compared to fructose [22]. In contrast, Cheng et al. [25] suggested that *L. plantarum* LPC-1 increased EPS production when grown in media containing sucrose. These results seem to indicate that it is possible to modulate EPS production, amount, structure and thus, properties by feeding specific LAB strains with different sugars. However, more data are needed to better understand the complex relationship between LAB strain, carbon source, EPS structure and activity. To fill this gap, the present study considered three LAB strains that are among the known EPS producer species. In particular, *L. bulgaricus* is one of the most commonly used starter species in cheese and yoghurt [26], while *L. paracasei* and *L. rhamnosus* are known for flavour formation in ripened cheeses and their potential health benefits [27,28].

Considering that LAB’s EPS production and structure strongly depend on several variables, each LAB was fed with five different sugars (fructose, glucose, lactose, maltose and sucrose) amongst the simple sugars found in different foods of animal and plant origin. The aim was to understand how and whether the variation of the carbon source supplied can affect strain growth, EPS production and chemical structure. Knowing which type and how much EPS is produced by varying the carbon source could be of great importance in defining the choices for EPS production for different uses.

## 2. Materials and Methods

### 2.1. Bacterial Strains, Growth Conditions and Media

Three wild LAB strains, namely *L. bulgaricus* 1932, *L. rhamnosus* 1019 and *L. paracasei* 2333, previously isolated from dairy products and belonging to the microbial collection of the Department of Food and Drug of the University of Parma (UPCC), were tested. All strains were stored as stock cultures at −80 °C in MRS broth (Oxoid, Basingstoke, UK), supplemented with 20% (*v*/*v*) glycerol until use. To obtain a fresh culture for use, bacteria were propagated twice (24 h at 37 °C) in anaerobiosis with a gas pack (Fisher Scientific, Rodano, Italy) in MRS broth.

Before use, cultures were harvested by centrifugation (10.000× *g*, 10 min at 4 °C) and washed three times with Ringer’s solution (Oxoid, Basingstoke, UK) to remove all media residue.

The washed cells were then transferred twice to modified MRS medium (mMRS), which was used as the base medium for EPS production. mMRS was prepared according to Degeest et al. [29] as follows: 30 g L^−1^ peptone (Oxoid, Basingstoke, UK), 12 g L^−1^ yeast extract (Merck, Darmstadt, Germany), 2 g L^−1^ K_2_HPO_4_ (Merck, Darmstadt, Germany), 5 g L^−1^ sodium acetate (Merck, Darmstadt, Germany), 2 g L^−1^ triammonium citrate (Merck, Darmstadt, Germany), 0.2 g L^−1^ MgSO_4_·7H_2_O (Sigma Aldrich, Darmstadt, Germany), 0.038 g L^−1^ MnSO_4_·H_2_O (Sigma Aldrich, Darmstadt, Germany), and 1 mL L^−1^ Tween 80 (Biolife, Monza, Italy) [29,30].

Each carbon source consisting of fructose (FRU), glucose (GLU), lactose (LAC), maltose (MAL) and sucrose (SUC), (Merck, Darmstadt, Germany), was fed to the microorganisms at a specific time point so that the bacteria could grow with each of the tested sugars as the sole carbon source. The sugar solutions were prepared as a concentrated water solution, sterilised separately from the medium, and then added to different bottles of mMRS to a final concentration of 5%.

The final cell concentration after duplicate propagation in mMRS was estimated by plate counting on MRS agar after 20 h of incubation (data not shown). After washing, the cells were inoculated into 6 mL of the five different aliquots of mMRS broths prepared with the five different sugars and cultured at 37 °C in anaerobiosis.

After 20 h of incubation, the cells were diluted to a final concentration of 1 × 10^7^ CFU/mL and used to inoculate: (i) 200 mL of media incubated in anaerobiosis at 37 °C and then used for the chemical analysis and (ii) 18 mL of media to determine growth behaviour.

### 2.2. Growth Behaviour of the Strains with Different Sugars

Eighteen ml of each of the five different mMRS was inoculated with *L. bulgaricus* 1932, *L. rhamnosus* 1019 and *L. paracasei* 2333, were divided equally among three sterlised BacTrac 4300^®^ measurement vials housed in the instrument BacTrac 4300^®^ Microbiological Analyzer System (SY-LAB, Neupurkersdorf, Austria) and incubated at 37 °C for 30 h. Each condition was analysed in triplicate, and two sets of analyses were performed.

During the 30-h incubation, the change in total conductance (M%) and capacitance (E%) was recorded every 10 min. The E% data were collected and fitted to the modified Gompertz equation according to Bancalari et al. [30,31] to obtain the kinetic parameters Rate and yEnd, which were used to describe the growth behaviour of the strains [31,32].

### 2.3. Impedance Measurement for the Detection of EPS Production

Information on EPS production was obtained from the impedance measurements for all tested samples that were studied. The E% value measured during the analysis was used to estimate the ability of the strains to produce EPS with the different sugars provided. As previously reported by Bancalari and colleagues [32], EPS production can be revealed by measuring the decrease in E% values. For this purpose, the parameter ΔE% was calculated as the difference between the maximum E% value and the value measured after 25 h of incubation.

**Figure 1:**
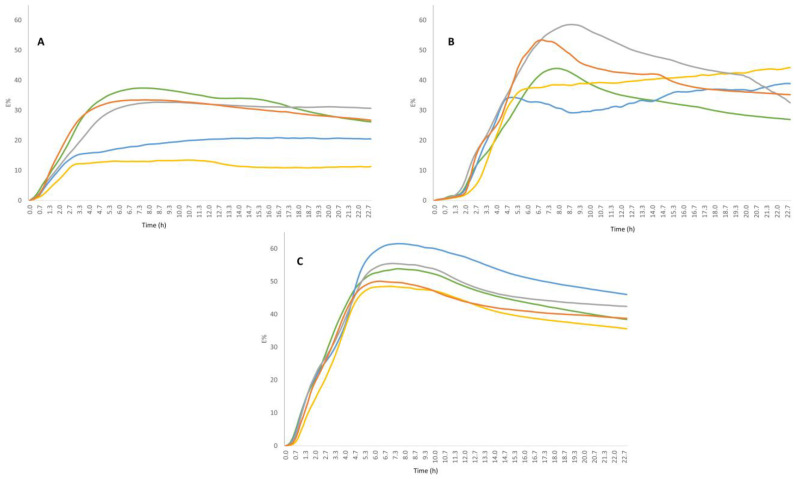
Impedometric curves of strains fed with the five different sugars (different colours). Each curve corresponds to the average curve from three independent experiments. (**A**) *L. rhamnosus* 1019, (**B**) *L. paracasei* 2333, (**C**) *L. bulgaricus* 1932.

The BacTrac 4300^®^ instrument, which was used to perform the impedometric analysis, allows the detection of bacterial activity in real time by measuring the decrease in impedance in an alternating current field. This is because during duplication, the viable cells of the bacteria break down sugars into smaller molecules that make the medium more conductive, resulting in a decrease in the total resistance and total impedance, which can thus be used as an indirect measure of bacterial metabolism [32]. During the incubation period, the instrument can register two specific impedance values for each individual measurement: (i) the conventional conductance (M value), which corresponds to the total medium impedance, and (ii) the capacitance value (E value), which is the measurement of the electrochemical double layer impedance near the electrodes. These two values, recorded simultaneously every 10 min and presented as relative changes compared to a baseline value, are expressed as M% and E% and visualised as a capacitance or conductance curve [31]. At the end of the analysis, all recorded capacitance data (E%) were used in three different ways: (i) to be fitted by the Gompertz equation to obtain the Rate and yEnd kinetic parameter, according to Bancalari et al. [30]; (ii) to build a graphical representation of the initial capacitance curve (Figure 1); (iii) to calculate the ΔE% parameter as an index of EPS production [32,33].

### 2.4. EPS Extraction and Quantification

The total amount of EPS produced by the strains in the five different mMRS was determined using the AOAC official enzymatic-gravimetric method for dietary fibre [34]. The analysis was performed using 20 mL of the sample in triplicate. Residual ash in the extracted fibres was determined by mineralisation at 550 °C for 5 h, while residual nitrogen was determined with a Kjeldahl system (DKL heating digestor and UDK 139 semiautomatic distillation unit, VELP SCIENTIFICA, Usmate Velate, Italy) and using a nitrogen-to-protein conversion factor of 6.25.

The same enzymatic-gravimetric method, with few modifications, was also used for EPS extraction, in order to allow further analysis of the chemical structure. After allowing 20 mL of the culture broth to react with heat-stable α-amylase, protease and amyloglucosidase in the amounts indicated by the official method, the EPS were precipitated by adding four volumes of 96% ethanol. The solution was then centrifuged at 3900 rpm and 4 °C for 30 min, and the pellet was finally dried overnight at 40 °C in an oven.

### 2.5. EPS Monosaccharide Composition by Gas Chromatography-Mass Spectrometry (GC-MS)

The composition of EPS monosaccharide was investigated using two different protocols. The first was for the detection of neutral and acid sugars, following a method previously proposed by Xia et al. with some modifications [35]. Here, 10 mg of EPS sample was dissolved in 3 mL of 2N trifluoroacetic acid (TFA) and hydrolysed at 110 °C for 2 h. Then, an aliquot of the solution was taken and mixed with 125 µL of 1.19 mg/mL phenyl-β-D-glucopyranoside, which was used as internal standard, and then evaporated by rotavapor. The dried hydrolysate obtained was washed with 1 mL of methanol, to remove the residual TFA, and evaporated again. Then, 1 mL of 0.5 M NH_4_OH was added to delactonise any acidic sugar lactones present in the sample and evaporated again by rotavapor. Finally, the dried hydrolysate was dissolved in 800 µL of dimethylformamide (DMF) and 200 µL of N,O-Bis(trimethylsilyl)trifluoroacetamide (BSTFA), the latter serving as derivatising agent. The reaction was kept at 60 °C for 1 h and then the derivatised sample was injected into the gas-chromatography.

The second protocol was used to detect amino sugars by acid hydrolysis with hydrochloric acid. Briefly, 10 mg of the sample was dissolved in 6 mL of 7 N HCl and kept at 110 °C for 4 h. Later, an aliquot was added to 125 µL of 1190 mg/L phenyl-β-D-glucopyranoside and they were evaporated together. As in the first method, 800 µL of DMF and 200 µL of BSTFA were then added, the reaction was kept at 60 °C for 1 h and the solution was ready for injection.

GC-MS analysis of monosaccharides was performed using a 6890 N gas chromatograph coupled to a 5973 N mass selective detector (Agilent technologies, Santa Clara, CA, USA). An SLB-5ms, 30 m × 0.25 mm, 0.25 µm thickness column (Supelco, Bellafonte, PA, USA) was used. The chromatogram was recorded in scan mode (40–500 *m*/*z*) with a programmed temperature from 60 °C to 270 °C. The initial temperature was 60 °C, held for 2 min, then increased to 160 °C at a rate of 10 °C/min, held isothermal for 5 min, increased to 220 °C at a rate of 10 °C/min and then kept for 5 min, increased to 270 °C at a rate of 20 °C/min and maintained for 5 min. Quantification was performed using a response factor, considering the area and concentration ratios between the internal standard (phenyl-β-D-glucopyranoside), and the following monosaccharides: D-glucose, D-fructose, D-galactose, D-mannose, D-rhamnose, D-ribose, D-xylose, D-fucose, D-galacturonic acid, D-glucuronic acid, D-glucosamine and D-galactosamine.

### 2.6. Evaluation of the EPS Molecular Weight by HPSEC-RID

The molecular weight of EPS produced by the selected strains was investigated by high-performance size-exclusion chromatography (HPSEC), using an Agilent 1260 Infinity II LC system equipped with a refractive index detector (RID) (Agilent, Santa Clara, CA, USA). EPS extracted from the culture broth (Paragraph 2.4) were dissolved into ultrapure water at a concentration of 10 mg/mL. The solutions were then filtered through a 0.45 µm membrane. An aqueous 50 mM NaCl solution was used as the mobile phase at a flow rate of 1 mL/min and a PL aquagel-OH MIXED-M column, 7.5 × 300 mm, 8 µm (Agilent, Santa Clara, CA, USA) was used to separate the different molecular weight fractions. The injection volume was set to 100 µL, the column temperature to 30 °C and the RID temperature to 35 °C. Standard pullulans of known molecular weight were purchased from Agilent (Santa Clara, CA, USA) and used for the calibration curve.

### 2.7. Statistical Analysis

All calculated parameters (amount of EPS produced, relative percentage of monosaccharides, ∆E%, yEnd, rate and percentage of EPS fractions with different molecular weights) were compared using the Pearson correlation using IBM SPSS version 21.0 software (SPSS Inc., Chicago, IL, USA). Significant correlations were considered for the values > 0.6 and <−0.6. In addition, a one-way analysis of variance (ANOVA) with Tukey’s post-hoc test, at a confidence level of 95% (*p*-value = 0.05), was performed using the same software to identify significant differences between the amounts of EPS produced in the different experiments.

## 3. Results and Discussion

### 3.1. Growth Behaviour of the Strains with Different Sugars

Impedometric curves obtained by continuously recording the change in E value (E%) during the incubation period are shown in Figure 1. It can be observed that the curves vary from strain to strain. *L. bulgaricus* 1932 showed less variability in terms of growth behaviour when fed with the different sugars. In contrast, *L. rhamnosus* 1019 and *L. paracasei* 2333 showed the greatest differences in growth behaviour depending on the sugar used. In particular, *L. rhamnosus* 1019 (Figure 1A) showed a lower growth capacity when fed with maltose and sucrose.

To quantify the differences, the E% data were fitted with the Gompertz equation, according to Bancalari et al. [31], and the kinetic parameters Rate and yEnd were used to describe the growth kinetics of the strains (Table 1). In particular, the Rate value describes the acidification speed, thus the higher the value, the faster the acidification, while yEnd represents the highest recorded impedance change, and is thus, interpreted as the maximum acidification capacity of the strain. Again, the higher the value, the greater the acidification capacity [31].

*L. paracasei* 2333 showed a similar growth capacity with comparable Rate values for four of the five sugars, with a lower value when cultivated in the presence of fructose (Table 1). However, the acidification capacity (Yend) was highly variable, ranging from the lowest values for fructose and maltose, to the highest values for lactose (Table 1, Figure 1b). Similarly, *L. bulgaricus* 1932 showed very similar Rate values for all sugars, but different acidification capacity depending on the sugar, ranging from 41.61 with sucrose to 53.59 with maltose (Table 1, Figure 1c). The lowest Rate and Yend values were obtained for *L. rhamnosus* 1019, indicating that the strain has difficulty developing under all conditions compared to the other strains, especially when fed with maltose and sucrose.

### 3.2. Quantification of EPS Production

**Figure 2:**
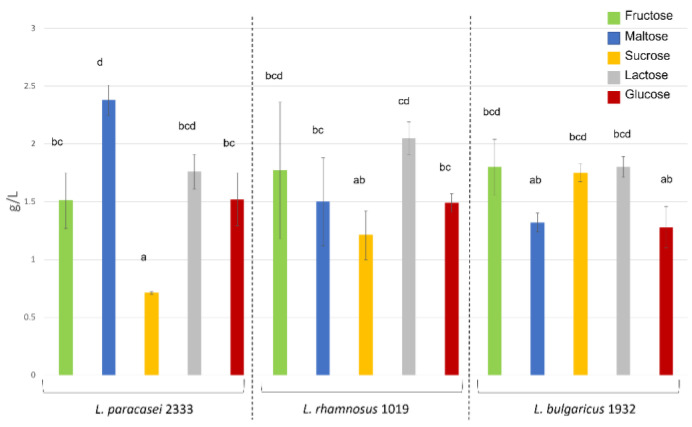
Amounts of EPS produced by the tested microorganisms with different carbon sources are given in g L^−1^ culture broth. Different letters above the bars indicate significant differences (one-way ANOVA, *p* < 0,05).

The ability of the strains to produce EPS was assessed by both impedometric analysis and the official gravimetric method. The E% value obtained from the impedometric analysis, which is the measure of the double-layer capacitance, was previously used by Bancalari and colleagues [32] to demonstrate the presence of released EPS during LAB growth [32]. The measured E% values were used to calculate the parameter ΔE%, which is the difference between the maximum E% value and the value recorded after 25 h of incubation (Table 1). In a previous work [33], strains causing a decrease in ∆E% value of more than 3, were assumed to be able to produce EPS. The data obtained by this method were compared with the gravimetric method normally used to quantify dietary fibre in complex samples. This method can achieve a higher degree of sample purification when used as an extraction method [36]. According to the impedometric method, *L. bulgaricus* 1932 was shown to be able to produce EPS from all sugars, with the highest peaks when fed with fructose and maltose and the lowest when fed with glucose (Table 1). The gravimetric method also showed no significant differences in the amounts of EPS for all sugars fed (Figure 2). *L. paracasei* 2333 analysed by impedometric method showed good EPS production in media containing fructose, lactose and glucose. On the other hand, EPS production was not observed in media containing sucrose and maltose as carbon sources (Table 1). Differently, using the gravimetric method, *L. paracasei* 2333 proved to be the best EPS producer when maltose was employed as carbon source, compared to the other two strains under the same conditions (Figure 2). However, when cultivated on sucrose, the significantly lowest amount of EPS was obtained for this strain. Moreover, a correlation can be observed between the quantification by gravimetric method and ∆E% for EPS production with the other three sugars, fructose, lactose, and glucose. The impedometric method showed that *L. rhamnosus* 1019 was only able to produce EPS when fed with fructose and glucose. However, the gravimetric method showed that the strain was able to produce EPS with any sugar, and the only significant difference was found in the experiments with lactose and sucrose, with the former resulting in a higher EPS content. The results obtained by the two methods agreed in 10 of 15 conditions tested. The greatest discrepancies were found when *L. rhamnous* 1019 was fed the disaccharides lactose, maltose, and sucrose, and when *L. paracasei* 2333 was fed with maltose and sucrose. The greatest agreements were highlighted for all strains when grown on fructose and glucose, and for *L. bulgaricus* 1932 under all conditions tested. These different results could be due to the different molecular composition of the EPS, which affects the electric measurement differently. 

However, impedometric analysis has been previously used to reveal EPS production, while a direct correlation with EPS has not yet been described. Based on these results, it can be concluded that the conductimetric estimate of EPS production can be used as a preliminary screening that needs to be confirmed by direct gravimetric analysis of EPS amount. Interestingly, both methods underlined that the maximum EPS amount was not achieved with sucrose as the sole carbon source, contrary to what has been previously reported in the literature [37,38]. Furthermore, these results show that different strains when fed with different sugars also behave differently in EPS production, highlighting the importance to consider the peculiarity and strain-specific attitudes when choosing the sugar to maximise the EPS production.

### 3.3. EPS Monosaccharide Composition

**Figure 3:**
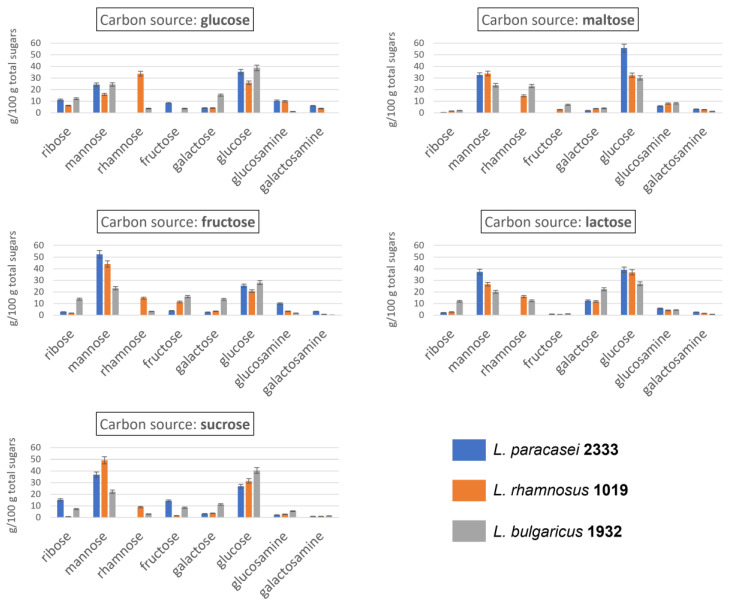
Monosaccharide composition (expressed as relative percentage) of EPS produced by three LAB strains fed five different sugars.

The monosaccharide composition of EPS produced by the three selected strains when fed with the five different sugars is shown in Figure 3. All three strains were able to produce EPS containing glucose, mannose, galactose, fructose, ribose, glucosamine and galactosamine as principal sugars. *L*. *rhamnosus* 1019 and *L. bulgaricus* 1932 produced EPS that also contained rhamnose, with *L. rhamnosus* 1019 able to incorporate this sugar into the EPS chain in amounts on average twice as large as the ones in EPS of *L. bulgaricus* 1932 (17.6% and 9%, respectively) when fed with all sugars except maltose. In contrast, *L. paracasei* 2333 was unable to incorporate rhamnose into the polysaccharide chain regardless of the carbon source with which it was fed, probably due to the absence of gene clusters encoding the production of this sugar [39]. Rhamnose was observed to be uncommon in EPS produced by lactobacilli in Zeidan et al. [14], which reports the presence of this sugar only for some strains of *L. bulgaricus* and *L. rhamnosus,* in agreement with our results. Apart from this marked difference, the other sugars were incorporated in all the EPS chains in similar, albeit variable, amounts. All three strains produced EPS consisting primarily of glucose and mannose, regardless of the carbon source added to the growth medium. The sum of mannose and glucose, expressed as a relative percentage of total sugars, ranged from 42% to 88%, with an average of 64%, suggesting that the EPS produced were mainly classifiable as glucomannans, despite the presence of other hexoses and pentoses [40,41,42]. Mannose seems to be present in high amounts when fructose was present as a carbon source, both as such and when present within sucrose chain. This might be supported by the fact that mannose and fructose are metabolically close, with only one metabolic step between them [43]. 

The results are consistent with the literature, which reports glucose, galactose, mannose, rhamnose, glucosamine and galactosamine as the most frequent monosaccharides in the EPS of LAB [20]. However, in some cases, fructose [44] and ribose [45] were also found as monosaccharides constituting the EPS chain produced by LAB. As concerns ribose, in the present study, considerable variations were found within the EPS produced by the same strain fed with different carbon sources: *L. paracasei* 2333 yielded ribose in amounts ranging from 0.5 to 15.3%, *L. rhamnosus* 1019 from 0.9 to 6.3% and *L. bulgaricus* 1932 from 2.1 to 13.8%. Fructose, on the other hand, varied between 0 and 14.3%, between 0 and 11.3% and between 1.1 and 16.1% in the EPS produced by *L. paracasei* 2333, *L. rhamnosus* 1019 and *L*. *bulgaricus* 1932, respectively, depending on the carbon source used. Galactose also showed considerable variability. It was particularly abundant in the EPS produced by *L. bulgaricus* 1932, with an average amount 2.5 times higher than that produced by the other two strains. In addition, the carbon source again influenced the quantities. In fact, a high amount was found when the three strains were grown on lactose, which is the only galactose-containing carbon source among those selected, and low amounts were found when maltose was used as feed. This is exactly the opposite of what was found in the study by Wang and Bi, who showed how maltose could increase the galactose content in EPS produced by *Lactobacillus kefiranofaciens* [46]. However, it must be emphasised that different bacterial species and strains may have completely different metabolic processes, and thus, different rules for EPS chain assembly. Finally, glucosamine and galactosamine were always found, but in relatively low amounts, ranging from 1.2 to 10.4% and from 0.1 to 6.1%, respectively. The presence of amino sugars in the EPS chain is of great importance, due to their characteristic electric charge. Indeed, when this latter is present on the EPS, it can lead to, depending on the ionic strength, an increase in the intramolecular repulsion forces and a consequent increase in the hydrodynamic volume and intrinsic viscosity [2].

### 3.4. EPS Molecular Weight (Mw)

The molecular weight of EPS produced in the different experiments was analysed by size exclusion chromatography coupled with a refractive index detector (HPSEC-RID). The results are shown in Table 2.

First, it can be noted that all the selected strains gave rise to HePS having different fractions of different Mw, as previously reported for LAB-deriving HePS [47]. 

*L. paracasei* 2333 produced EPS that were always very similar to each other, regardless of the carbon source used. In particular, three different fractions with Mw between 10 and 200 kDa occurred in all experiments, and always in very similar proportions. The two most abundant fractions had an Mw variable in the range of 10–65 kDa and accounted for 84–95% of the total EPS. When sucrose was used as the carbon source, another very small fraction, with an Mw of less than 10 kDa, was detected and accounted for 12% of the total EPS. These results are in overall agreement with a previous study by Hamet’s group, which tested five *L. paracasei* strains [48].

Both *L. rhamnosus* 1019 and *L. paracasei* 2333 produced EPS consisting mainly of medium Mw fractions, which together accounted for 78–94% of the total EPS for four out of five sugars. The most peculiar case was again found when *L. rhamnosus* 1019 was fed sucrose, as an abundant fraction of low Mw, accounting for 32% of the total (and putatively reported to be 4 kDa) was detected. Moreover, in two experiments in which sucrose and lactose were added as the sole carbon source, even a high-Mw fraction (>500 kDa) was detected, albeit in small amounts. These results are in partial agreement with a previous study showing that another *L. rhamnosus* strain was able to produce EPS of very different sizes depending on the carbon source, although in this case they turned out to be much bigger [49]. This suggests that this structural peculiarity is mainly related to the selected strain. *L. bulgaricus* 1932 was the strain that differed the most compared to the others: although it also always produced EPS with abundant medium Mw fractions, they were less abundant, and their sum was 43–80%. The presence of the high-Mw fraction was also detected, in amounts ranging from 15 to 18%, when the strain was grown on all carbon sources except maltose. When maltose, glucose and lactose were added to the growth medium, the EPS produced by *L. bulgaricus* 1932 were also characterised by the smallest fraction (<10 kDa), which accounted for 9, 30 or 36% of the total EPS. 

Overall, these results indicate that the Mw profile of EPS is clearly dependent on the bacterial strain. However, in several cases, the carbon source may also strongly influence this characteristic, although not always in a predictable and constant manner. As far as we know, there are no clear reports in the literature on how and why bacterial strains use different sugar sources to build EPS with different sizes. For this reason, much work remains to be done, but this work contributes to our understanding of this relationship. In general, the Mw of HePS from LAB reported in the literature, varies between 10^4^ and 10^6^ Da [50]. Our values fall within this range, with the sole exception of fraction 5, whose Mw is <10 kDa. It should be noted that many authors perform an ultrafiltration step (10 kDa cut-off) before analysing the Mw of the EPS considered [51], preventing the detection of this fraction. However, the presence of EPS with low Mw can be considered an element of further enhancement of the producing strains, since these EPS have been described as more effective in terms of antioxidant activity [52], although the structure–function relationship of this property is still debated among scientists. Conversely, the high Mw fractions found in some samples, especially those derived from *L*. *bulgaricus* 1932, could have an interesting potential for technological and functional activities related to viscosity. Indeed, the positive correlation between the Mw of EPS and the induced viscosity is now well known, similar to the correlation between an increase in viscosity and better cholesterol-lowering and antimicrobial properties [20].

### 3.5. Correlation Analysis between Factors Involved in EPS Production

To elucidate the complex factors involved in EPS production and the related molecular features, a correlation analysis was performed between all the data collected in this study.

Table 3 shows the correlation matrix between EPS chemical characteristics (monosaccharide composition and molecular weight distribution), EPS production and strain growth behaviour, independently from the feed sugars. Coloured cells represent data with a negative (red) or positive (green) correlation score higher than 0.6, indicating a strong association between the variables studied. A first consideration that arises from the analysis is the lack of correlation between the impedometric and molecular data. Indeed, the acidification speed (Rate), the maximum acidification capacity (yEnd) and the ability to produce EPS (∆E > 3) do not correlate with any molecular characteristic of EPS. Not surprisingly, Rate and yEnd are positively correlated. ∆E% does not correlate with total EPS (g/L), confirming the limitations of the impedometric analysis to predict EPS production in some cases, as reported in paragraph 3.2, probably due to the specific molecular composition/charge of some EPS. Apart from the positive correlation between glucosamine and galactosamine, indicating that the inclusion of positively charged sugars into the EPS chain occurs using both aminosugar epimers, no other significant correlation was found between monosaccharide composition. The most interesting data that emerged is the correlation between some EPS fractions with a certain molecular weight and the presence of some monosaccharides. These significant correlations allow us to derive more information about the distribution of monosaccharides in the EPS of different molecular weights. Specifically, fraction 1 is positively correlated with galactose, which means that EPS with a molecular weight from 130 to 200 kDa contained this monosaccharide. The presence of galactose was highest in the EPS produced by *L. bulgaricus* 1932 (Figure 3), which were also the EPS with the highest Mw. Therefore, it can be suggested that only *L. bulgaricus* is characterised by the ability to produce EPS with a larger molecular size and that these EPS contain galactose. A positive correlation is observed between EPS fraction 3 and fructose, which, in turn, is abundant in EPS when fructose sources are present in the feed sugars. Since this fraction has Mw between 40 and 65 kDa, this result suggests that LAB produces medium to small EPS when fructose is the available carbon source. EPS fraction 4 is negatively correlated with galactose and positively correlated with glucosamine and galactosamine. This fraction includes EPS with Mw between 8 and 25 kDa. These correlations indicate the presence of charged HePS in this Mw range.

## 4. Conclusions

By analysing the EPS production of three different LAB strains fed with five different sugars, we found that the EPS production differed significantly among both the different strains and carbon sources. Variability in the monosaccharide composition and molecular weight of EPS was also found. For example, *L. bulgaricus* 1932 showed the most constant EPS production when fed with all sugars, but it produced EPS that differed greatly in monosaccharide composition and molecular weight, which, in turn, could have different effects on the nutritional and technological properties of EPS. Therefore, it would be of great importance to choose the most suitable carbon source to produce EPS for the intended use. In addition, it could be very interesting to study the production of EPS using LAB strains mainly known for their technological properties, as it was the case of *L. paracasei* 2333 which is currently studied. These results open new perspectives for the use of LAB to produce tailored EPS.

## Figures and Tables

**Table 1:** Kinetic parameters (Rate), (yEnd) and (∆E%) obtained by impedometric analysis of the tested strains.

Strain	Rate	yEnd	∆E%
*L. paracasei* 2333 FRU	8.84	33.46	16.95
*L. paracasei* 2333 MAL	12.58	33.6	−8.21
*L. paracasei* 2333 SUC	11.3	40.18	−5.75
*L. paracasei* 2333 LAC	11.25	46.7	25.19
*L. paracasei* 2333 GLU	12.26	41.27	16.2
*L. rhamnosus* 1019 FRU	8.16	32.47	11.19
*L. rhamnosus* 1019 MAL	2.49	19.58	−1.77
*L. rhamnosus* 1019 SUC	4.3	11.54	1.71
*L. rhamnosus* 1019 LAC	5.95	31.16	1.9
*L. rhamnosus* 1019 GLU	8.89	30.25	6.72
*L. bulgaricus* 1932 FRU	11,34	45.92	15.27
*L. bulgaricus* 1932 MAL	10.59	53.59	15.42
*L. bulgaricus* 1932 SUC	12.94	41.61	12.59
*L. bulgaricus* 1932 LAC	10.36	47.66	12.79
*L. bulgaricus* 1932 GLU	11.3	43.11	10.65

**Table 2:** Molecular weight profile (expressed as relative percentage of total chromatographic area) of EPS produced by three LAB strains, fed with five different carbon sources.

		% of Total Peak Area				
Strain	Carbon Source	Fraction 1 (>500 kDa)	Fraction 2 (130–200 kDa)	Fraction 3 (40–65 kDa)	Fraction 4 (10–25 kDa)	Fraction 5 (<10 kDa)
*L. paracasei* 2333	Fructose	−	7	38	56	−
	Maltose	−	5	42	53	−
	Sucrose	−	4	44	40	12
	Lactose	−	6	41	52	−
	Glucose	−	9	37	54	−
*L. rhamnosus* 1019	Fructose	−	10	42	47	−
	Maltose	−	6	36	57	−
	Sucrose	4	10	25	29	32
	Lactose	5	17	36	42	−
	Glucose	−	8	34	57	−
*L. bulgaricus* 1932	Fructose	16	4	61	19	−
	Maltose	−	11	32	48	9
	Sucrose	15	5	47	32	−
	Lactose	18	3	24	20	36
	Glucose	15	3	29	23	30

**Table 3:** Correlation matrix of factors involved in EPS production and composition.

Correlations	EPS g/L	Ribose	Mannose	Rhamnose	Fructose	Galactose	Glucose	GlucosAmine	Galactosamine	Rate	yEnd	∆E	Fraction 1	Fraction 2	Fraction 3	Fraction 4	Fraction 5
EPS g/L	1	−0.382	−0.180	−0.032	−0.346	0.230	0.503	0.076	0.126	0.122	0.05	0.05	0.145	0.151	0.229	0.084	−0.369
Ribose	−0.382	1	−0.468	−0.268	0.549 *	0.464	−0.218	−0.340	−0.191	0.497	0.484	0.095	0.555 *	−0.534 *	0.229	−0.560 *	0.294
Mannose	−0.180	−0.468	1	−0.371	0.002	−0.489	−0.168	−0.071	−0.038	−0.392	−0.504	−0.096	−0.463	0.127	−0.038	0.243	−0.009
Rhamnose	−0.032	−0.268	−0.371	1	−0.286	−0.088	−0.395	0.279	−0.022	−0.427	−0.169	−0.085	−0.155	0.440	−0.368	0.193	−0.003
Fructose	−0.346	0.549 *	0.002	−0.286	1	−0.084	−0.388	−0.325	−0.291	0.328	0.337	0.115	0.152	−0.210	0.672 **	−0.283	−0.195
Galactose	0.230	0.464	−0.489	−0.088	−0.084	1	0.031	−0.457	−0.474	0.244	0.48	0.386	0.850 **	−0.311	−0.081	−0.726 **	0.457
Glucose	0.503	−0.218	−0.168	−0.395	−0.388	0.031	1	−0.024	0.215	0.319	0.032	−0.271	0.034	−0.105	0.057	0.079	−0.115
Glucosamine	0.076	−0.340	−0.071	0.279	−0.325	−0.457	−0.024	1	0.848 **	−0.044	−0.044	0.205	−0.541 *	0.219	−0.165	0.778 **	−0.472
Galactosamine	0.126	−0.191	−0.038	−0.022	−0.291	−0.474	0.215	0.848 **	1	0.061	−0.14	0.047	−0.585 *	0.161	−0.070	0.762 **	−0.484
Rate	−0.141	0.334	−0.150	−0.284	0.292	−0.314	0.170	0.336	0.369	1	0.792 **	0.337	−0.033	−0.300	0.161	0.130	−0.131
yEnd	−0.189	0.400	−0.389	−0.083	0.488	−0.017	−0.051	0.250	0.089	0.792 **	1	0.563 *	0.140	−0.225	0.258	0.003	−0.190
∆E	−0.024	0.123	−0.216	−0.011	0.227	0.312	−0.218	0.218	−0.073	0.337	0.563 *	1	0.309	−0.024	0.047	−0.147	−0.030
Fraction1	0.145	0.555 *	−0.463	−0.155	0.152	0.850 **	0.034	−0.541 *	−0.585 *	0.272	0.341	0.216	1	−0.431	0.069	−0.893 **	0.483
Fraction2	0.151	−0.534 *	0.127	0.440	−0.210	−0.311	−0.105	0.219	0.161	−0.459	−0.304	−0.045	−0.431	1	−0.203	0.342	−0.272
Fraction3	0.229	0.229	−0.038	−0.368	0.672 **	−0.081	0.057	−0.165	−0.070	0.373	0.25	0.084	0.069	−0.203	1	−0.015	−0.669 **
Fraction4	0.084	−0.560 *	0.243	0.193	−0.283	−0.726 **	0.079	0.778 **	0.762 **	−0.173	−0.232	−0.08	−0.893 **	0.342	−0.015	1	−0.667 **
Fraction5	−0.369	0.294	−0.009	−0.003	−0.195	0.457	−0.115	−0.472	−0.484	−0.087	−0.018	−0.076	0.483	−0.272	−0.669 **	−0.667 **	1

“Fraction” represents different EPS groups with different molecular mass: Fraction 1 (>500 kDa), Fraction 2 (130–200 kDa), Fraction 3 (40–65 kDa), Fraction 4 (8–25 kDa) and Fraction 5 (<10 kDa). ** Correlation is significant at a 0.01 level. * Correlation is significant at a 0.05 level. Coloured cells represent data with a negative (red) or positive (green) correlation score higher than 0.6, indicating a strong association between the variables studied. The darker the colour the stronger the correlation.

## Data Availability

The datasets generated for this study are available on request to the corresponding author.
